# Virtual Reality as an Emerging Methodology for Leadership Assessment and Training

**DOI:** 10.3389/fpsyg.2018.01658

**Published:** 2018-09-11

**Authors:** Mariano Alcañiz, Elena Parra, Irene Alice Chicchi Giglioli

**Affiliations:** Instituto de Investigación e Innovación en Bioingeniería, Universitat Politècnica de València, Valencia, Spain

**Keywords:** leadership, neuroscience, virtual reality, presence, stealth assessment

## Abstract

In developed countries, companies are now substantially reliant on the skills and abilities of their leaders to tackle a variety of complex issues. There is a growing consensus that leadership development training and assessment methods should adopt more holistic methodologies, including those associated with the emotional and neuroendocrine aspects of learning. Recent research into the assessment of leadership competencies has proposed the use of objective methods and measurements based on neuroscience. One of the challenges to be faced in the development of a performance-based methodology to measure leadership skills is how to generate real-life situations with triggers that allow us to study management competencies under controlled laboratory conditions. A way to address this question is to take advantage of virtual environments to recreate real-life situations that might arise in performance-based assessments. We propose virtual reality (VR) as a very promising tool to observe various leadership related behavioral patterns during dynamic, complex and realistic situations. By seamlessly embedding assessment methods into virtual learning environments, VR can provide objective assessment methods with high ecological validity. VR also holds unlimited opportunities for leadership training providing subjects with intelligent tutoring systems that adapts situations in real time according to the observed behaviors.

## Introduction

In an increasingly globalized world, companies need to commit to important changes to the strategies they use to develop their human resources in terms of knowledge and leadership (Thorpe et al., 2010).

The effectiveness of current leadership development techniques has been challenged (Haines, 2009; Beer et al., 2016), and it is suggested that new skills should be developed, especially the ability to cope with stress and uncertainty, while maintaining a positive focus on the future (Nicholson, 2009). There is a growing consensus that leadership development training should adopt more holistic methodologies, including those associated with the emotional and neuroendocrine aspects of learning (Boyatzis et al., 2006).

A growing number of organizational behavior and human resource management (OBHRM) scholars are calling for greater attention to be paid to the influence that brain processes have on behavior and attitudes in the workplace (Ashkanasy et al., 2014). This has led to the emergence of a multidisciplinary field, Organizational Neuroscience (ON), which uses information from the brain to allow researchers and practitioners to generate new knowledge in the OB and HRM fields.

Several ON techniques have been proposed for use in this field, such as psychophysiological signals processing (Miller et al., 2007; Sequeira et al., 2009; Glöckner and Herbold, 2011; Durantin et al., 2014), brain activity monitoring (Boyatzis and McKee, 2011; Ernst et al., 2013; Hannah et al., 2013) and social physics (Choudhury and Pentland, 2002).

These techniques have been used in the design of laboratory-based experiments with controlled stimuli that exclude some variables present in real life situations. Thus, the ecological validity of these experiments is quite limited. Conversely, it is not easy to study human responses in real life situations because of the experimenter’s inability to totally control the stimuli involved in the experience.

In the present article, we propose the use of virtual reality (VR) as a new approach to elicit leadership competencies, skills, and behaviors. Virtual reality has emerged as a promising technology that can overcome the problems of achieving valid results from real life scenarios. VR provides the opportunity to simulate real life situations, including social situations, triggering embodied experiences in which the body, environment and brain are in close relationship. Consequently, behaviors, attitudes and beliefs can be transferred from reality to virtuality, and vice versa, in a spontaneous, unconscious, unaware manner, allowing the creation and modulation of life like scenarios with high ecological validity, while maintaining high experimental control both of the presentation of the stimuli and of the capturing of behavioral performance.

## Neuroscience and Organizational Behavior

In recent times, a growing interest has developed in “looking” inside the brain to seek solutions to problems that have not traditionally been addressed by neurosciences. We are, thus, witnessing an expansion of the use of neurosciences by academics and researchers. One discipline that is showing a special interest in the use of neurosciences is organizational behavior and human resources management (OBHRM). The current model of “brain-directed man” posits that emotions override cognitive processes and hence impact on behaviors. The sources of motivation rely on complex structures based on neural substrates. (Ghadiri, 2012).

Traditionally, most theories of OBHRM are based on a model of the human mind that assumes that humans can think and verbalize accurately about their attitudes, emotions and behaviors (Simon, 1976; Brief, 1998). Thus, to date, most of the theoretical constructs used in OB and HRM are based on explicit measures such as self-reports and interviews. However, recent advances in neuroscience have demonstrated that most of the brain processes that regulate our emotions, attitudes and behaviors are not conscious. That is, they are implicit processes that, in contrast to explicit processes, humans cannot verbalize (Barsade et al., 2009; George, 2009; Becker et al., 2011).

To address these limitations, a growing number of OBHRM scholars are calling for greater attention to be paid to the influence that implicit processes have on behavior and attitudes in the workplace (Ashkanasy, 2013; Ashkanasy et al., 2014; Healey and Hodgkinson, 2015; Ward et al., 2015). This has led to an emerging multidisciplinary field, ON, which uses information from the brain to give researchers and practitioners a better understanding of the disciplines of OB and HRM (Waldman and Balthazard, 2015).

Traditionally, in OBHRM research, the most widely used and validated assessment methods are self-report questionnaires, interviews and projective measures. Self-report measures can be conditioned by “social desirability effects,” which can lead to inaccurate accounts of behaviors, attitudes and beliefs (Paulhus, 1991). Secondly, there may be different interpretations of specific self-report items (e.g., the definition of “to be creative”), resulting in unreliability and poorer validity (Lanyon and Goodstein, 1997). Thirdly, some self-reporting questions need people to possess overt knowledge of their dispositions (Schmitt, 1994), and this is not always the case.

In stark contrast, by using both implicit and explicit measures, ON can help OBHRM scholars develop more complete and integrated theories of human behavior in the workplace.

Several implicit measuring techniques have recently been proposed. Cortisol levels have been measured to study workplace stress related to low personal control (Fox et al., 1993; Dickerson and Kemeny, 2004; Miller et al., 2007).

Skin conductance level has been successfully measured as an indicator of implicit processes such as stress, affective arousal and cognitive processing (Nikula, 1991; Sequeira et al., 2009).

Heart variability (HV) has been used for the implicit measurement of complex phenomena such as emotional intelligence (Craig et al., 2009), stress (Thayer et al., 2012) and cognitive load (Durantin et al., 2014).

Eye tracking (ET) constitutes a very interesting measure of subconscious brain processes that show correlations with information processing in risky decisions (Glöckner and Herbold, 2011), empathy (Wang et al., 2006) and problem solving (Knoblich et al., 2001).

Electroencephalograms (EEG), functional magnetic resonance imaging (fMRI) and functional near infrared spectroscopy (fNIRS) are being used as implicit measures to monitor brain activity. EEG measures have been proven to correlate with leadership assessment and team engagement (Waldman et al., 2013), inspirational leadership assessment (Waldman et al., 2011) and decision-making (Hannah et al., 2013).

Functional magnetic resonance imaging is being used as a direct measure of brain activity related to implicit processes such as social exclusion, emotional engagement and emotion regulation (Boyatzis et al., 2014).

Recent studies, using fNIRS, into decision-making under pressure (Tsujii and Watanabe, 2010) and into decision-making processes in approach-avoidance theories (Ernst et al., 2013), are highly relevant for OB and HRM.

A recent discipline that is bringing a new focus onto implicit measures is social physics. Social physics integrates big data techniques with custom sensors to study how to increase cooperation, productivity, creativity and wellness in the workplace (Khan, 2017).

These implicit measurement techniques have mostly been used in the design of experiments carried out in laboratory settings with controlled stimuli; but they have also been used to observe participants’ behaviors in real scenarios.

On the one hand, when experiments are undertaken in the laboratory, potentially influential variables affecting the subject’s responses can be closely monitored. On the other hand, in the laboratory the controlled stimuli given to the subject often do not include variables that are present in real life situations. Thus, the ecological validity of these methodologies is quite limited. However, it is not easy to study human responses in real situations because of the experimenter’s inability to totally control the stimuli involved in the experience.

## Virtual Reality and Organizational Neuroscience

Virtual reality can be seen as a computer-based 3D synthetic environment able to simulate real experiences in which subjects can interact as if they were in the real world (Pratt et al., 1995). Various technological devices (visual, auditory and haptic), tracking systems (e.g., Head Mounted Displays – HMD) with a high degree of accuracy in stimuli reproduction can create a sense of presence, the subjective psychological state of “being there” in the virtual world, leaving the perception of technological mediation behind (Biocca, 1997; Slater, 2009; Pillai et al., 2013).

Virtual reality can track various responses, both at behavioral and neuro-physiological level. Through VR low-cost body-motion tracking systems, it is possible to measure the user’s behavior in real time during the virtual experience. These systems allow tracking of non-verbal expressions during VR-mediated interactions. In addition, low-cost ET systems integrated into VR goggles allow us to analyze gaze activity, providing highly valuable information about cognitive states. Furthermore, several miniaturized wearables can be used to obtain psycho-physiological signals, which, after processing, provide valuable indirect sources of information related to brain correlates of leadership competencies. VR is capable of fine-grained recording of implicit measures: integrating them with self-reported descriptions of the experience can build a more comprehensive and complete model of human responses. Neuroscience and psychology scholars find it difficult, or even impossible, to achieve such a degree of multisensory stimulation and embodied interaction using other methods.

It has been shown that the neural mechanisms that humans have when immersed in virtual environments are very similar to the mechanisms that originate in real life (Tarr and Warren, 2002; Alcañiz et al., 2009). A recent wide-ranging review of the existing literature on VR and social cognitive neuroscience (Loomis et al., 1999) reported the effective use of VR in several domains, such as cognitive neuropsychological assessment, affective induction and social psychology (Parsons, 2015). In cognitive neuropsychological assessment, VR can measure the complexity of responses with high correspondence with daily situations (Elkind et al., 2001; Parsons et al., 2007; Climent and Bánterla, 2011; Henry et al., 2012; Iriarte et al., 2012; Jovanovski et al., 2012a,b; Renison et al., 2012; Lalonde et al., 2013; Díaz-Orueta et al., 2014).

Social neuroscientists are adding social interaction variables to the development of virtual interactive environments. Studies of neuroimaging and psychophysiology during virtual social situations are investigating the brain regions involved in the interpretation of other people’s face and eye movements (Schilbach et al., 2006, 2010; Carter and Pelphrey, 2008; Slater et al., 2010; Rotge et al., 2014).

Many studies from the past decade found VR to be efficacious, easy to use, safe, and contributing to training capabilities and decision making in several fields like healthcare (Fertleman et al., 2018; Walsh et al., 2018), education (Freina and Ott, 2015), and industry (Sportillo et al., 2015; Lawson et al., 2016), among others.

However, until very recently, VR has not been used in ON methodologies to enhance the stimuli control and ecological validity of implicit measures in OBHRM. We propose VR as a very promising tool to examine various behavioral patterns during dynamic, complex and realistic situations.

## Virtual Stealth Assessment Method for Leadership Research

A recently developed and promising research area for tracking behavioral performance and evaluating leadership attributes and skills is “stealth assessment.” The technique is based on the concept that assessment methods can seamlessly be embedded into virtual learning environments. The term stealth assessment was coined by Valery Shute to describe the process of handling data obtained during game playing to inconspicuously track students’ knowledge, skills and attributes (Shute, 2009). More specifically, the stealth assessment method presents the possibility of assessing various behaviors related to particular skills and attributes, thus providing indirect real-time evaluations (Mislevy et al., 2003). To make valid inferences from the assessments, the stealth method is supported by evidence-centered design (ECD), a conceptual reference framework that can be used to develop assessment models (Shute, 2011). In accordance with ECD, three conceptual models must be developed before the game to be used in the experiment is created: competency, evidence and task models. The competency model aims to describe the set of skills and competences to be assessed. The evidence model aims to show the behaviors provoked in response to a problem that could demonstrate the skill sets individuated in the competency model. At the same time, the evidence model proposes the statistical *a priori* probabilities of the relationship between behaviors and skills. Finally, the task model aims to develop tasks or situations able to elicit those behaviors related to the set of skills. Stealth assessment can also be defined as a performance-based assessment where what is being assessed is latent (Rupp et al., 2010).

In accordance with the stealth assessment method, competency identification aims to describe the set of skills and competences that should be assessed. When the main competencies for assessment are identified (the latent components), the evidence model aims to reveal the real behaviors that can elicit these competencies. For each theoretical competency, various behaviors are identified and described, and these should appear when the subject is presented with a particular problem. The last phase in the generation of the theoretical framework is the creation of tasks or situations able to elicit those competency related behaviors that the experimenter wishes to assess.

## Conclusion

Companies experience moments of uncertainty, risk and chaos. One of the main features of the current socio-economic context is innovation and change. This has led to a paradigm shift in organizational management from a taylorist mechanistic model to a more complicated paradigm based on complex non-linear relationships. For this reason, today, company leaders must possess or develop a set of diverse and complementary horizontal skills such as problem-solving, status-quo questioning, creativity, self-management and team leadership, among others, that are crucial for the actual organizational paradigms.

As for leadership assessment methods, traditional techniques based on explicit measures using questionnaires, interviews and self-reports, are being increasingly questioned. In a recent article titled “How to get a job at Google”, Lazlo Bock, senior vice president of people operations at Google, stated that *“Assessing leadership skills is much more difficult, and it is something that is almost impossible to find out in an interview (Friedman, 2014)”.*

Traditional OBHRM models were based on the measurement of explicit processes through questionnaires and interviews. Since the mid-1990s, OBHRM academics have been taking into account the influence that implicit brain processes have on personal relationships in business and decision-making. Implicit activities, emotions, automatic body responses and unconscious brain processes shape the way we think, feel and act at work.

For this reason, methodologies and techniques, under the name of ON, are emerging. They combine neuroscience and OBHRM competences to better understand, train and measure the horizontal capabilities required by the modern leader. ON techniques to measure implicit processes are being applied in real situations, with the consequent loss of control of the contextual variables that can affect the measurements. They are also being used in laboratories, where it is impossible to reproduce the complex daily company context, which results in low ecological validity. To address these limitations, we propose that VR technologies, coupled with the stealth assessment method, provide a possible means of examining behavioral patterns during dynamic, complex and realistic situations.

Company leaders should certainly consider VR and the stealth assessment method for leadership assessment and training research activities. In the near future, we envisage that specific computer software will be designed for leadership assessment and training activities that will unobtrusively provide reliable and objective metrics for leadership-related competencies. In accordance with the stealth assessment method, we propose a methodological framework based on this ECD able to indirectly assess leadership competences and skills in action and in real-time during a virtual experience. Indeed, the stealth method, more than traditional methods, allows the assessment of various competencies or constructs through the development of numerous problems or tasks, providing performance or behavior indices.

Virtual reality is a crucial experimental tool in the management field that investigates new objective-based methods for leadership assessment. With VR based assessment tools, it is possible to recreate real-life situations where the presented stimuli and the effects of neural mechanisms can be measured in real time.

We have previously emphasized that the special interest being shown in implicit measures does not invalidate the more traditional techniques based on explicit measures. Moreover, we believe that an appropriate balance between implicit and explicit measures can substantially improve the models of the human mind used in OBHRM.

We believe that, soon, OBHRM specialists and consultants will use VR systems to enable leaders better to develop their capabilities. As the participant will face virtual situations similar to those that she or he has to face daily in the workplace, it will be possible to obtain real-time metrics of psychological constructs very relevant to any leader. This real-time measurement capacity will foster the development of neurofeedback architectures that will, in turn, modify the situations presented in real time, in order to provide leaders with intelligent tutoring systems to allow them to improve their capabilities. If only explicit measures were used, it would not be possible to develop neurofeedback methods that would allow the virtual environment to adapt in real time to the emotional and cognitive states of the user; the sense of presence in the virtual environment would be negatively affected when subjects verbalized their internal brain processes and this, in turn, would negatively affect the ecological validity of the measures and training carried out.

The development of these systems is, from a technological point of view, already fully viable. Advances in software technologies in areas such as VR, advanced signal processing, cognitive systems and artificial intelligence, make such systems a reality. Indeed, the huge advances in low cost VR hardware devices allow us to affirm that, in the very near future, these systems will be routinely used in companies, outside of laboratory settings.

In the near future, we hypothesize that VR applications will used routinely to predict leadership attributes and skills. One possible prototype application is below described.

The application could create specific narratives story-telling formed by different situations. Each virtual situations would be developed following the stealth assessment methodology described before, and would be based in one of the transformational leadership competences. Each virtual situation would contain virtual characters with specific psychological traits relevant with the different dimensions of each leadership competence. The virtual characters would interact with the subject by mean of spoken dialogs and facial and body gestures. In accordance with the virtual situations the roles among the characters could change but the final decision-making will always depend on the participant of the study.

Through the independent variables of each situation (type of situations presented along the virtual script, personality type of characters, and emotional charge of situations) it would be possible to trigger different behaviors in the subject that conforms the dependent variables. The dependent variables to be measured in each situation would be the decision taken by the subject, the response time for taking the decision and the subject’s gaze dynamics measured using eye-tracking techniques.

We will illustrate an example of situation related with empathy. Empathy capabilities could be assessed facing the subject to a situation presenting a dilemma between helping or not a dependent character. The decision taken by the subject can be used as a metric of empathy. Moreover, using ET techniques, if the subject’s visual pattern would be constant attending to the face during a direct interaction versus attending the environment, this value of empathy would be even greater. That is, each decision-making in each situation could give a concrete value (based on the weight of the correlation with traditional techniques as questionnaires) to a previously defined skill. In the same way, a visual pattern directly linked to each situation or each role of the character would also offer a specific weight to each skill.

We conclude by addressing ethical aspects of the use of these future technologies. Obviously, because of their implicit nature, these systems will provide a large amount of sensitive data. Therefore, these data should be treated with care and with the guarantee that at all times they will be used only for positive purposes.

Nonetheless, with this proviso, we believe that the use of implicit measuring techniques integrated into virtual environments will greatly improve our knowledge of why leaders feel and act in the way they do.

## Ethics Statement

Before participating in the study, each participant was provided with written information about the study and required to give written consent for inclusion in the study. The study received ethical approval by the Ethical Committee of the Polytechnic University of Valencia.

## Author Contributions

All authors have significantly contributed to the manuscript according to their particular scientific competences. MA made substantial contributions to the manuscript conception and supervised the entire work. IC and EP participated in drafting the manuscript. All the authors have given their final approval to the version to be submitted and any revised version.

## Conflict of Interest Statement

The authors declare that the research was conducted in the absence of any commercial or financial relationships that could be construed as a potential conflict of interest.

## References

[B1] Alcañiz M. Rey B. Tembl J. Parkhutik V. (2009). A neuroscience approach to virtual reality experience using transcranial Doppler monitoring. *Presence* 18 97–111. 10.1162/pres.18.2.97

[B2] Ashkanasy N. M. (2013). Neuroscience and leadership: take care not to throw the baby out with the bathwater. *J. Manage. Inq.* 22 311–313. 10.1177/1056492613478519

[B3] Ashkanasy N. M. Becker W. J. Waldman D. A. (2014). Neuroscience and organizational behavior: avoiding both neuro-euphoria and neuro-phobia. *J. Organ. Behav.* 35 909–919. 10.1002/job.1952

[B4] Barsade S. G. Ramarajan L. Westen D. (2009). “Implicit affect in organizations,” *Research in Organizational Behavior*, Vol. 29 (New York, NY: Jai Press Inc), 135–162 10.1016/j.riob.2009.06.008

[B5] Becker W. J. Cropanzano R. Sanfey A. G. (2011). Organizational neuroscience: taking organizational theory inside the neural black box. *J. Manage.* 37 933–961. 10.1177/0149206311398955

[B6] Beer M. Finnström M. Schrader D. (2016). *Why Leadership Training Fails and What to Do About It. Harvard Business Review, (October).* Available at: https://hbr.org/2016/10/why-leadership-training-fails-and-what-to-do-about-it

[B7] Biocca F. (1997). The cyborg’s dilemma: progressive embodiment in virtual environments. *J. Comput. Med. Commun.* 3 28–57.

[B8] Boyatzis R. McKee A. (2011). Neuroscience and leadership: the promise of insights. *Ivey Bus. J.* 75 1–3.

[B9] Boyatzis R. E. Rochford K. Jack A. I. (2014). Antagonistic neural networks underlying differentiated leadership roles. *Front. Hum. Neurosci.* 8:114. 10.3389/fnhum.2014.00114 24624074 PMC3941086

[B10] Boyatzis R. E. Smith M. L. Blaize N. (2006). Developing sustainable leaders through coaching and compassion. *Acad. Manage. Learn. Educ.* 5 8–24. 10.5465/amle.2006.20388381

[B11] Brief A. P. (1998). *Attitudes in and Around Organizations.* Thousand Oaks, CA: Sage.

[B12] Carter E. J. Pelphrey K. A. (2008). Friend or foe? Brain systems involved in the perception of dynamic signals of menacing and friendly social approaches. *Soc. Neurosci.* 3 151–163. 10.1080/17470910801903431 18633856

[B13] Choudhury T. Pentland A. (2002). “The sociometer: a wearable device for understanding human networks,” in *CSCW’02 Workshop: Ad hoc Communications and Collaboration in Ubiquitous Computing Environments*, Kauai.

[B14] Climent G. Bánterla F. (2011). *Nesplora Classroom, Ecological Assessment of Attentional Processes, Theoretical Manual.* Donostia, Spain: Nesplora.

[B15] Craig A. Tran Y. Hermens G. Williams L. M. Kemp A. Morris C. (2009). Psychological and neural correlates of emotional intelligence in a large sample of adult males and females. *Pers. Ind. Diff.* 46 111–115. 10.1016/j.paid.2008.09.011

[B16] Díaz-Orueta U. Garcia-López C. Crespo-Eguílaz N. Sánchez-Carpintero R. Climent G. Narbona J. (2014). AULA virtual reality test as an attention measure: convergent validity with conners’ continuous performance test. *Child Neuropsychol.* 20 328–342. 10.1080/09297049.2013.792332 23638628

[B17] Dickerson S. S. Kemeny M. E. (2004). Acute stressors and cortisol responses: a theoretical integration and synthesis of laboratory research. *Psychol. Bull.* 130 355–391. 10.1037/0033-2909.130.3.355 15122924

[B18] Durantin G. Gagnon J. F. Tremblay S. Dehais F. (2014). Using near infrared spectroscopy and heart rate variability to detect mental overload. *Behav. Brain Res.* 259 16–23. 10.1016/j.bbr.2013.10.042 24184083

[B19] Elkind J. S. Rubin E. Rosenthal S. Skoff B. Prather P. (2001). A simulated reality scenario compared with the computerized Wisconsin Card Sorting Test: an analysis of preliminary results. *Cyber Psychol. Behav.* 4 489–496. 10.1089/109493101750527042 11708728

[B20] Ernst L. H. Plichta M. M. Lutz E. Zesewitz A. K. Tupak S. V. Dresler T. (2013). Prefrontal activation patterns of automatic and regulated approach-avoidance reactions—a functional near-infrared spec- troscopy (fNIRS) study. *Cortex* 49 131–142. 10.1016/j.cortex.2011 22036575

[B21] Fertleman C. Aubugeau-Williams P. Sher C. Lim A. N. Lumley S. Delacroix S. (2018). A discussion of virtual reality as a new tool for training healthcare professionals. *Front. Public Health* 6:44. 10.3389/fpubh.2018.00044 29535997 PMC5834832

[B22] Fox M. L. Dwyer D. J. Ganster D. C. (1993). Effects of stressful job demands and control on physiological and attitudinal outcomes in a hospital setting. *Acad. Manage. J.* 36 289–318. 10125121

[B23] Freina L. Ott M. (2015). “A literature review on immersive virtual reality in education: state of the art and perspectives,” in *Proceedings of eLearning and Software for Education* (Bucharest: eLSE).

[B24] Friedman T. L. (2014). *How to Get a Job at Google.* Available at: https://www.nytimes.com/2014/02/23/opinion/sunday/friedman-how-to-get-a-job-at-google.html

[B25] George J. M. (2009). The illusion of will in organizational behavior research: nonconscious processes and job design. *J. Manage.* 35 1318–1339. 10.1177/0149206309346337

[B26] Ghadiri A. (2012). *Neuroleadership A Journey Through the Brain for Business Leaders.* Berlin: Springer-Verlag.

[B27] Glöckner A. Herbold A. K. (2011). An eye-tracking study on information processing in risky decisions: evidence for compensatory strategies based on automatic processes. *J. Behav. Decis. Mak.* 24 71–98. 10.1002/bdm.684

[B28] Haines S. (2009). Bankrupt leadership development? *Training* 46:64.

[B29] Hannah S. T. Balthazard P. A. Waldman D. A. Jennings P. Thatcher R. (2013). The psychological and neurological bases of leader self-complexity and effects on adaptive decision-making. *J. Appl. Psychol.* 98 393–411. 10.1037/a0032257 23544481

[B30] Healey M. P. Hodgkinson G. P. (2015). “Toward a theoretical framework for organizational neuroscience,” in *Organizational Neuroscience (Monographs in Leadership and Management* Vol. 7 eds Waldman D. A. Balthazard P. A. (Bingley: Emerald Group Publishing Limited), 51–81. 10.1108/S1479-357120150000007002

[B31] Henry M. Joyal C. C. Nolin P. (2012). Development and initial assessment of a new paradigm for assessing cognitive and motor inhibition: the bimodal virtual-reality Stroop. *J. Neurosci. Methods* 210 125–131. 10.1016/j.jneumeth.2012.07.025 22897988

[B32] Iriarte Y. Diaz-Orueta U. Cueto E. Irazustabarrena P. Banterla F. Climent G. (2012). AULA—advanced virtual reality tool for the assessment of attention: normative study in Spain. *J. Atten. Disord.* 20 542–568. 10.1177/1087054712465335 23239784

[B33] Jovanovski D. Zakzanis K. Campbell Z. Erb S. Nussbaum D. (2012a). Development of a novel, ecologically oriented virtual reality measure of executive function: the multitasking in the city test. *Appl. Neuropsychol. Adult* 19 171–182. 10.1080/09084282.2011.643955 23373603

[B34] Jovanovski D. Zakzanis K. Ruttan L. Campbell Z. Erb S. Nussbaum D. (2012b). Ecologically valid assessment of executive dysfunction using a novel virtual reality task in patients with acquired brain injury. *Appl. Neuropsychol. Adult* 19 207–220. 10.1080/09084282.2011.643956 23373607

[B35] Khan S. M. (2017). “Multimodal behavioral analytics in intelligent learning and assessment systems,” in *Innovative Assessment of Collaboration*, eds von Davier A. A. Zhu M. Kyllonen P. C. (Berlin: Springer International Publishing), 173–184.

[B36] Knoblich G. Ohlsson S. Raney G. E. (2001). An eye movement study of insight problem solving. *Mem. Cognit.* 29 1000–1009. 10.3758/BF03195762 11820744

[B37] Lalonde G. Henry M. Drouin-Germain A. Nolin P. Beauchamp M. H. (2013). Assessment of executive function in adolescence: a comparison of traditional and virtual reality tools. *J. Neurosci. Methods* 219 76–82. 10.1016/j.jneumeth.2013.07.005 23867080

[B38] Lanyon R. I. Goodstein L. D. (1997). *Personality Assessment*, 3rd Edn New York, NY: Wiley.

[B39] Lawson G. Salanitri D. Waterfield B. (2016). Future directions for the development of virtual reality within an automotive manufacturer. *Appl. Ergon.* 53 323–330. 10.1016/j.apergo.2015.06.024 26164106

[B40] Loomis J. M. Blascovich J. J. Beall A. C. (1999). Immersive virtual environment technology as a basic research tool in psychology. *Behav. Res. Methods Instrum. Comput.* 31 557–564. 10.3758/BF03200735 10633974

[B41] Miller G. E. Chen E. Zhou E. S. (2007). If it goes up, must it come down? Chronic stress and the hypothalamic-pituitary-adrenocortical axis in humans. *Psychol. Bull.* 133 25–45. 10.1037/0033-2909.133.1.25 17201569

[B42] Mislevy R. J. Almond R. G. Lukas J. F. (2003). A brief introduction to evidence-centered design. *ETS Res. Rep. Ser.* 2003 i–29. 10.1002/j.2333-8504.2003.tb01908.x

[B43] Nicholson N. (2009). Leading in tough times. *Bus. Strat. Rev.* 20 38–41. 10.1111/j.1467-8616.2009.00596.x

[B44] Nikula R. (1991). Psychological correlates of nonspecific skin conductance responses. *Psychophysiology* 28 86–90. 10.1111/j.1469-8986.1991.tb03392.x 1886966

[B45] Parsons T. D. (2015). Virtual reality for enhanced ecological validity and experimental control in the clinical, affective and social neurosciences. *Front. Hum. Neurosci.* 9:660. 10.3389/fnhum.2015.00660 26696869 PMC4675850

[B46] Parsons T. D. Bowerly T. Buckwalter J. G. Rizzo A. A. (2007). A controlled clinical comparison of attention performance in children with ADHD in a virtual reality classroom compared to standard neuropsychological methods. *Child. Neuropsychol.* 13 363–381. 10.1080/13825580600943473 17564852

[B47] Paulhus D. L. (1991). “Measurement and control of response bias,” in *Measures of Personality and Social Psychological Attitudes*, eds Robinson J. P. Shaver P. R. Wrightsman L. S. (Cambridge, MA: Academic Press), 1759 10.1016/B978-0-12-590241-0.50006-X

[B48] Pillai J. S. Schmidt C. Richir S. (2013). Achieving presence through evoked reality. *Front. Psychol.* 4:86. 10.3389/fpsyg.2013.00086 23550234 PMC3581802

[B49] Pratt D. R. Zyda M. Kelleher K. (1995). Virtual reality: in the mind of the beholder. *IEEE Comput.* 28 17–19.

[B50] Renison B. Ponsford J. Testa R. Richardson B. Brownfield K. (2012). The ecological and construct validity of a newly developed measure of executive function: the Virtual Library Task. *J. Int. Neuropsychol. Soc.* 18 440–450. 10.1017/S1355617711001883 22339816

[B51] Rotge J. Y. Lemogne C. Hinfray S. Huguet P. Grynszpan O. Tartour E. (2014). A meta-analysis of the anterior cingulate contribution to social pain. *Soc. Cogn. Affect. Neurosci.* 10 19–27. 10.1093/scan/nsu110 25140048 PMC4994851

[B52] Rupp A. A. Gushta M. Mislevy R. J. Shaffer D. W. (2010). Evidence-centered design of epistemic games: measurement principles for complex learning environments. *J. Technol. Learn. Assess.* 8.

[B53] Schilbach L. Wilms M. Eickhoff S. B. Romanzetti S. Tepest R. Bente G. (2010). Minds made for sharing: initiating joint attention recruits reward-related neurocircuitry. *J. Cogn. Neurosci.* 22 2702–2715. 10.1162/jocn.2009.21401 19929761

[B54] Schilbach L. Wohlschlaeger A. M. Kraemer N. C. Newen A. Shah N. J. Fink G. R. (2006). Being with virtual others: neural correlates of social interaction. *Neuropsychologia* 44 718–730. 10.1016/j.neuropsychologia.2005.07.017 16171833

[B55] Schmitt N. (1994). Method bias: the importance of theory and measurement. *J. Organ. Behav.* 15 393–398. 10.1002/job.4030150504

[B56] Sequeira H. Hot P. Silvert L. Delplanque S. (2009). Electrical autonomic correlates of emotion. *Int. J. Psychophysiol.* 71 50–56. 10.1016/j.ijpsycho.2008.07.009 18723054

[B57] Shute V. J. (2009). Simply assessment. *Int. J. Learn. Media* 1 1–11. 10.1162/ijlm.2009.0014

[B58] Shute V. J. (2011). Stealth assessment in computer-based games to support learning. *Comput. Games Instr.* 55 503–524.

[B59] Simon H. A. (1976). *Administrative Behavior: A Study of Decision-making Processes in Administrative Organization.* New York, NY: Macmillan.

[B60] Slater M. (2009). Place illusion and plausibility can lead to realistic behaviour in immersive virtual environments. *Philos. Trans. R. Soc. B Biol. Sci.* 364 3549–3557. 10.1098/rstb.2009.0138 19884149 PMC2781884

[B61] Slater M. Spanlang B. Sanchez-Vives M. V. Blanke O. (2010). First person experience of body transfer in virtual reality. *PLoS One* 5:e10564. 10.1371/journal.pone.0010564 20485681 PMC2868878

[B62] Sportillo D. Avveduto G. Tecchia F. Carrozzino M. (2015). “Training in VR: a preliminary study on learning assembly/disassembly sequences,” in *Augmented and Virtual Reality: Second International Conference, AVR 2015*, eds De Paolis L. T. Mongelli A. (Lecce: Springer), 332–343. 10.1007/978-3-319-22888-4_24

[B63] Tarr M. J. Warren W. H. (2002). Virtual reality in behavioral neuroscience and beyond. *Nat. Neurosci.* 5 1089–1092. 10.1038/nn948 12403993

[B64] Thayer J. F. Åhs F. Fredrikson M. Sollers J. J. Wager T. D. (2012). A meta-analysis of heart rate variability and neuroimaging studies: implications for heart rate variability as a marker of stress and health. *Neurosci. Biobehav. Rev.* 36 747–756. 10.1016/j.neubiorev.2011.11.009 22178086

[B65] Thorpe R. Gold J. Mumford A. (2010). *Gower Handbook of Leadership and Management Development.* Farnham: Gower Publishing Company.

[B66] Tsujii T. Watanabe S. (2010). Neural correlates of belief-bias reasoning under time pressure: a near-infrared spectroscopy study. *Neuroimage* 50 1320–1326. 10.1016/j.neuroimage.2010.01.026 20080190

[B67] Waldman D. A. Balthazard P. A. (2015). “Organizational neuroscience,” in *Organizational Neuroscience (Monographs in Leadership and Management*, eds Waldman David A. Balthazard A. Pierre (Bingley: Emerald Group Publishing Limited), 7.

[B68] Waldman D. A. Balthazard P. A. Peterson S. J. (2011). Leadership and neuroscience: can we revolutionize the way that inspirational leaders are identified and developed? *Acad. Manage. Perspect.* 25 60–74. 10.5465/amp.25.1.60

[B69] Waldman D. A. Wang D. Stikic M. Berka C. Balthazard P. A. Richardson T. Maak T. (2013). “Emergent leadership and team engagement: an application of neuroscience technology and methods,” in *Academy of Management Proceedings* Vol. 2013 (Briarcliff Manor, NY: Academy of Management), 12966.

[B70] Walsh C. Lydon S. Byrne D. Madden C. Fox S. O’connor P. (2018). The 100 most cited articles on healthcare simulation: a bibliometric review. *Simulat. Healthcare* 13 211–220. 10.1097/SIH.0000000000000293 29613918

[B71] Wang H. Chignell M. Ishizuka M. (2006). “Empathic tutoring software agents using real-time eye tracking,” in *Proceedings of the 2006 Symposium on Eye Tracking Research & Applications* (New York, NY: ACM), 73–78.

[B72] Ward M. K. Volk S. Becker W. J. (2015). “An overview of organizational neuroscience,” in *Organizational Neuroscience*, eds Waldman D. A Balthazard P. A. (Bingley: Emerald Group Publishing Limited), 17–50. 10.1108/S1479-357120150000007001

